# The efficacy of open molds in controlling tinnitus

**DOI:** 10.1016/S1808-8694(15)30081-1

**Published:** 2015-10-19

**Authors:** Gisele Munhoes dos Santos Ferrari, Tanit Ganz Sanchez, Maria Elisabete Bovino Pedalini

**Affiliations:** a M.S in Sciences –Medical School – University of São Paulo, clinical speech and hearing therapist, audiologist and TRT specialist; b Associate Professor of Otorhinolaryngology – University of São Paulo Medical School; c PhD. Speech and Hearing Therapist – University of São Paulo Medical School – Department of Otorhinolaryngology

**Keywords:** hearing aids, randomized controlled trials, hearing loss, tinnitus

## Abstract

Hearing aids may be a option to improve tinnitus and hearing loss. **Aim:** to evaluate tinnitus after one month use of BTE hearing aids with open molds and pressure vent molds in patients with symmetric sensorineural hearing loss. **Methods:** 50 patients seen at our Tinnitus Clinic who presented bilateral tinnitus and hearing loss underwent a randomized blind crossover clinical trial: 26 first used BTE hearing aids with open molds, and the remaining 24 first used pressure vent molds. After 30 days using the first mold and a wash-out period, the type of earmold was changed and was applied for another 30-day-period. Tinnitus evaluation was done qualitatively (improved, unchanged and worsened) and quantitatively (variation on a numeric scale from 0 to 10). **Results:** 82% of the cases reported improvement of tinnitus with at least one type of earmold; there was no significant difference in the reduction of discomfort due to tinnitus in the quantitative and qualitative evaluations. Although similar tinnitus control was obtained with both methods, 66% of the patients preferred the open mold. **Conclusion:** In a short-term evaluation improvement of tinnitus by the use of hearing aids does not depend on earmold ventilation.

## INTRODUCTION

About 90% of tinnitus patients also have some degree of hearing loss.^1^, ^2^, ^3^, ^4^, ^5^ Tinnitus significantly worsens the quality of life in 15% to 25% of cases, affecting sleep, concentration, emotional balance and social activities.^6^, ^7^, ^8^ Hearing loss may also significantly impair daily living by imposing limits in communication.

Hearing aids are routinely used to minimize the effects of hearing loss;^9^ its use requires adapting an adequate earmold for each user. Earmolds should be chosen according to the audiological and anatomical needs of users and the electroacoustic characteristics of hearing aids to attain the benefits of adaptation.^10^

Many patients, however, are not comfortable with the occlusion of the external acoustic meatus (EAM) by hearing aid earmolds. This occlusion effect is characterized clinically by a blocked ear sensation, annoyance with one’s own voice and with chewing noises,^11^, ^12^ all of which are significantly amplified by bone conduction of sound.^13^ In some cases EAM occlusion may also worsen the perception of tinnitus,^14^, ^15^ a fairly frequent clinical finding.

A relatively simple way to reduce the occlusion effect produced by earmolds on the EAM is to open a vent hole in parallel with the original earmold hole. Venting allows amplified low frequency sounds to escape, providing relief from the blocked ear sensation, a relative increase of the response to treble sounds and improved sound quality.^11^ Vents may have various diameters according to the needs of patients. One millimeter vents generally are sufficient to equalize pressure; attenuation of the occlusion effect may require larger diameters. A non-occlusive or vented earmold is recommended if a drastic reduction of amplification at frequencies below 1000 Hz is required.^10^

Published papers that have defended the use of hearing aids for controlling tinnitus generally focus on evaluating the masking effect produced by hearing aid-amplified ambient sounds on tinnitus. These papers have rarely specified the diameter of the vent hole, the type of earmold, or the manner by which these aids were adapted to patients.

With the advent of Tinnitus Retraining Therapy (TRT) in 1990,^16^ hearing aid adaptation and sound generators with vented earmolds started to be used for the long-term (about 18 months) treatment of tinnitus. The true influence of the earmold vent size on the short-term treatment of tinnitus with hearing aids is not yet known.

In our clinic we have observed that some tinnitus patients with sensorineural hearing loss, which had not improved satisfactorily with poorly vented hearing aids, attained better control of tinnitus with larger earmold vents. This finding motivated this study, where the main aim was to assess the response of tinnitus patients to behind-the-ear (BTE) hearing aids with two types of earmold vents (vented earmold and pressure vent), in patients with mild to severe sensorineural symmetric hearing loss, after one month of hearing aid use.

Secondary aims were to check possible variations in the response of tinnitus to earmold vent types according to the audiometric configuration of hearing loss, and to assess the response of hearing loss to both type of earmold vents, correlating the tinnitus response with the hearing loss response.

## SERIES AND METHODS

We conducted a randomized blinded crossover clinical trial in which the type of hearing aid earmold was exchanged during the study. Approval was obtained from the Committee for the Analysis of Research Projects of the Clinical Hospital of the Sao Paulo University Medical School (HCFMUSP) (CAPPesq, protocol 738/02); funding was provided by the FAPESP Support for Research Grants (process number 02/09199-0).

The sample included 50 subjects, 28 (56%) women and 22 (44%) men, aged between 25 and 89 years, with a mean age of 64.4 years and a standard deviation (SD) of 13.1 years, registered in our Tinnitus Research Group.

Inclusion criteria were as follows: the presence of bilateral constant tinnitus in adult subjects of both genders; the presence of bilateral symmetrical sensorineural hearing loss of any etiology in which hearing aids were indicated; awareness of the trial requirements and signing of a free informed consent form.

Exclusion criteria were as follows: bilateral asymmetrical sensorineural hearing loss defined as a 15 dB difference in two or more frequencies; profound sensorineural hearing loss in two or more frequencies; mixed hearing loss; and a medical contraindication or refusal to try hearing aids.

Hearing loss was assessed by pure tone audiometry before the study as one of the sample selection criteria. Upon inclusion, 37 (74%) patients presented hearing loss with a downward sloping audiometric configuration and 13 (26%) patients had hearing loss with concomitant low frequency involvement.

After inclusion, hearing was further assessed by free-field audiometry at 500, 1000, 2000 and 4000 Hz with and with no hearing aids after 30 days of household experience with each type of earmold. Benefits due to hearing aids were assessed by the following tests:

- Quantitative assessment using a numeric scale (NS) from 0 to 10 to measure annoyance due to hearing loss; this test was applied before the trial and 30 days after household experience with each type of earmold.
- Qualitative assessment using a closed question applied at 30 days of testing each type of earmold: “What happened to your hearing loss?” where the answer was chosen from the following options: “improved”, “unaltered “, or “worsened”.

Both hearing and tinnitus (annoyance) were assessed using quantitative and qualitative methods.

### Procedures

The pre-inclusion assessment was conducted by the otorhinolaryngologist in charge of the Tinnitus Research Group based on a medical and audiological evaluation protocol used routinely in the clinic. After inclusion patients were monitored by the speech therapist in charge of the trial and randomly allocated to one of the following two groups:

Group 1 received binaural adaptation of hearing aids with vented earmolds followed by earmolds with pressure vents.
Group 2 received binaural adaptation of hearing aids with pressure-vented earmolds followed by vented earmolds.

The patients were evaluated to classify annoyance due to tinnitus and hearing loss in a 0 to 10 NS before receiving a hearing with the first earmold. Each group of patients was instructed to use the hearing aids for 30 days. After this period a “blinded” to the trial speech therapist assessed all patients with free-field audiometry with and with no hearing aids, and conducted the quantitative and qualitative tests for tinnitus and hearing loss.

**Figure 1:**
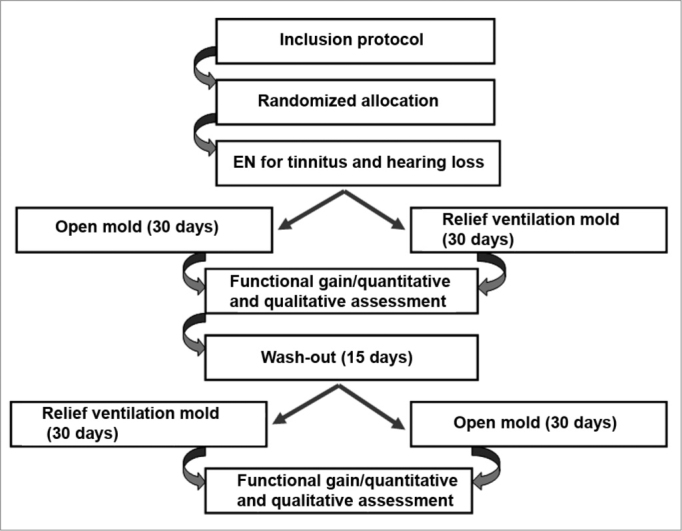
Sequence of procedures done in each patient.

After this first stage the patients were kept free of hearing aids for fifteen days (wash-out period) to eliminate any effect that the first earmold might have on the second earmold. Patients then used the second type of earmold for another 30 days, after which a similar evaluation was done by the same speech therapist “blinded” to the trial (Figure 1).

The earmolds were simple invisible rigid acrylic earmolds made by the same prosthetic professional in two vent sizes as follows:

Pressure vent: a 1mm diameter hole in parallel to the earmold hole, with the aim of balancing EAM and atmospheric air pressure;

Vented earmold (maximum vent): a 4 mm diameter hole that drastically reduced the amplification of frequencies below 1000 Hz.

The patients were fitted with GnResound Danalogic model 163 BTE hearing aids to avoid any influence of hearing aid type on outcomes. The hearing aids had the following main features to allow flexibility for different audiometric configurations: 6 channels, 3 comfort programs, acoustic feedback digital suppressor, wide dynamic range compression, suppression of ambient noise and directional digital amplification.

At the end of the trial, patients that responded favorably to hearing aid tests when using at least one of the earmold types were given the hearing aids free of charge.

Statistical analysis included categorical variable frequencies, and measurement of the central tendency and dispersion for quantitative variables. The Wilcoxon test was used for quantitative measurements and the McNemar chi-square test was used for qualitative data. Spearman’s (f) correlation coefficient was also used. The statistical significance level was p ≤ 0.05.

## RESULTS

The mean tinnitus annoyance score before the trial went from 1 to 10 points, with a mean 7.1 points (SD: 2.2). The hearing loss annoyance score varied from 0 to 10 points, with a mean 6.2 points (SD: 2.4).

The vented earmold was used initially in 26 patients (52%), followed by the pressure vent after the wash-out period. The remaining 24 patients (48%) started the trial using pressure vented earmolds followed by the vented earmold in the second part of the trial.

There was no NS score difference in the quantitative assessment of tinnitus following the use of both vent sizes. The mean annoyance score was 3.7 with the vented earmold and 3.9 with the pressure vent (p=0.96). The qualitative assessment revealed that 41 patients (82%) reported improvement from tinnitus with at least one type of earmold. No significant difference was observed in the effect of earmold venting on annoyance due to tinnitus (McNemar chi-square=0.00; p=1.00).

The quantitative assessment of hearing loss showed that annoyance due to hearing loss tended to regress when using pressure vents compared to vented earmolds, although this trend was not statistically significant (p=0.11). The qualitative assessment showed that 46 patients (92%) reported improvement of hearing with both types of earmolds; 2 patients (4%) improved only with the pressure vent. No significant difference was found in the earmold venting effect over annoyance due to hearing loss (McNemar chi-square=2.00; p=0.16).

Patients were regrouped into two other groups to check whether the response of tinnitus to venting varied with audiometric configurations, as follows:

Grupo D included patients with downward sloping hearing loss (n = 37, 74% of the sample).
Grupo P included patients with flat configuration hearing loss and those patients in which lower frequencies were also affected (n = 13; 26% of the sample).

There was no significant quantitative NS score difference in annoyance due to tinnitus in group D patients. Patients in group P tended to be less annoyed from the tinnitus when using the pressure vent (p = 0.08). There was no significant qualitative difference in annoyance due to tinnitus for both types of earmolds in both groups.

The quantitative assessment of hearing loss showed that annoyance due to hearing loss tended to regress when using pressure vents compared to vented earmolds in group P patients. The qualitative assessment showed no significant difference in annoyance due to hearing loss when using both types of earmolds in both groups.

**Chart 1:**
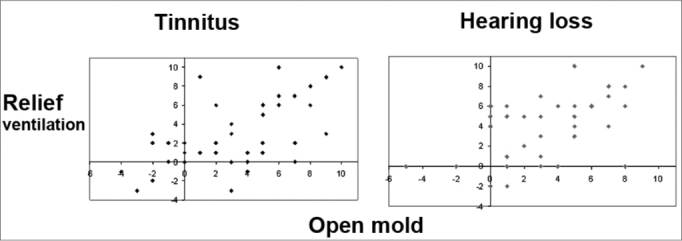
Correlation between measurements of variation of annoyance due to tinnitus and hearing loss assessed by a NS.

There was a positive correlation between the variation of measurements of annoyance due to tinnitus and hearing loss assessed by the NS when using vented earmolds and pressure vents (Chart 1). The regression of annoyance due to tinnitus and hearing loss with one type of earmold occurred in a similar proportion to the regression of annoyance with the other type of earmold.

Vented earmolds were preferred by 66% of patients, regardless of having started the trial using vented earmolds or pressure vents. Twenty-six group D patients (70.3%) preferred vented earmolds; seven group P patients (53.9%) also preferred this type of earmold.

Functional gain was significantly higher when using pressure vents at 500 Hz and 1000 Hz (p=0.03) in group P patients.

Of the 50 patients involved in this trial, four (8%) refused hearing aids after the study; 3 were dissatisfied with the results and 1 was unable to handle the prosthesis adequately. These patients returned the hearing aids at the end of the trial and continue to be monitored at the Tinnitus Research Group of the HCFMUSP.

## DISCUSSION

Hearing loss may be the most important factor in the prevalence of tinnitus; both findings are age-related.^17^, ^18^, ^19^, ^20^, ^21^ According to a survey done by the National Center for Health Statistics in 1987,^22^ hearing loss is the third and tinnitus is the tenth most frequent chronic condition in the elderly. There is a strong correlation between the incidence of tinnitus and presbyacusis, which is a frequent cause of age-related hearing loss.^23^

Our study sample consisted mostly of elderly patients with ownward sloping sensorineural hearing loss (74%). Sheldrake and Hazell (1991) and Santos et al. (1999) have also shown that this type of audiogram occurs in about 60% of tinnitus patients.^24^, ^25^

Tinnitus tends to be mild and intermittent in 80% of cases, which does not produce significant negative consequences for individuals; these persons generally do not seek medical care for this reason. Hearing loss generally progresses slowly - as opposed to tinnitus, which usually appears abruptly - and may not be perceived in its initial stages. It becomes significant when communication is affected.^26^ The repercussion of both findings according to the NS in our sample was 7.1 points for annoyance due to tinnitus and 6.2 points for annoyance due to hearing loss. Our patients came from a reference ENT unit and as such tended to present higher annoyance levels compared to the population at large.

The use of sound devices to relieve tinnitus dates from Aristotle, who toyed with the idea that a stronger sound could mask a weaker sound.21 When a patient also presents hearing loss, sound stimulation has to be done through hearing aids;^27^ in such cases amplification of ambient sound may partially reduce or eliminate tinnitus-associated annoyance.^28^, ^29^, ^30^, ^31^, ^32^

Von Wedel et al. (1989) compared the benefits of hearing aids and tinnitus maskers in 74 patients during three years and found total or partial masking of tinnitus with hearing aids in 80% of cases.33 Similar results were also presented by Moura et al. (2004), who showed improvement of tinnitus in 87.2% of 47 cases; in this study, there was total masking of tinnitus in 51% of patients after 3 to 8 months of hearing aid use.34 Folmer et al. (2002) found that hearing aids used during nine months reduced the intensity of tinnitus in 69% of 123 cases.31 Kiessling (1980) and Surr and Mueller (1985) reported satisfactory masking of tinnitus in 50% of patients wearing hearing aids for a few weeks.^35^, ^36^

During our 30-day study period we were able to see improvements in tinnitus due to partial or total masking in 82% of our sample. We considered this time-frame as a habituation period, without losing our focus which was to assess the benefit brought by hearing aids in the short-term improvement of tinnitus.^37^

Automatic masking of tinnitus in 82% of cases may be considered a satisfactory result compared to other forms of tinnitus therapy. We believe, however, that relief only occurs while using hearing aids; patients again perceive tinnitus as soon as the hearing aid is removed.^21^, ^38^ The participants of this trial are being followed up to monitor the long-term effect of hearing aids, as improved auditory thresholds may lead to plastic changes in the central nervous system (CNS) that may only be perceived after a prolonged period of peripheral stimulation.

Neuroscience investigation shows that CNS plasticity requires longer time periods, similar to the premise of Tinnitus Retraining Therapy (TRT), which is based on the neurophysiological model described by Jastreboff in 1990.16 In TRT the required time for habituation to tinnitus is about 18 months.

Adaptation of hearing aids in TRT is indicated for patients that consider hearing loss a significant problem in their lives. The impact of hearing loss on the life of a patient is more important this his or her audiometric configuration.^38^, ^39^ In our study patients that were little annoyed from their hearing loss were also encouraged to participate in the clinical trial to assess the effect of hearing aids on tinnitus and communication.

In TRT, non-occlusion of the EAM when adapting instruments (sound generators or hearing aids) is essential to assure the passage of ambient non-amplified low frequencies to the EAM, which favors the habituation process to tinnitus.40 Decreased annoyance due to tinnitus would be expected in the long-term for patients using vented earmolds. In our study we did not take into account the habituation process; we considered only the tinnitus masking effect produced by simple amplification of ambient sound. Furthermore, adaptation of hearing aids for TRT is only part of a complex process that also includes therapeutic counseling so that patients may understand the generation, detection and perception mechanisms of tinnitus. The main function of hearing aids in TRT it to act on the tinnitus detection process, enriching the patient’s sound environment.^39^

An advantage of this approach is habituation to the perception of tinnitus; although a prolonged period may be required, definitive improvements results from plastic changes in the CNS, where patients cease perceiving tinnitus in most situations of daily life. 71% to 87.5% of TRT category 2 patients (clinically significant tinnitus and hearing loss), treated with therapeutic counseling and hearing aids, reported definitive improvement of tinnitus after one year of treatment.^41^, ^42^

### Occlusion effect

Vented earmolds have been used frequently in hearing aid adaptation for downward sloping configuration hearing loss since the 1970s. A study on earmold preference (vented or unvented) showed that 83.3% of patients with sloping hearing loss preferred vented earmolds.^43^ Kuk (1991) also assessed preference for earmold types (vented or unvented) in nine patients after using hearing aids with each type of earmold during three months and found that vented earmolds maximize hearing aid acceptance by improving the quality of one’s own voice and by increasing the sharpness of sound.^44^

Dillon (2001) and Voogdt (2002) reported the occlusion effect in patients with hearing loss under 40 dB NA at low frequencies (downward sloping configuration), and suggested that a 2mm vent could avoid occlusion, although vent sizes 3mm or above might be needed.^12^, ^13^ In our trial we compared the effects of two vent sizes, 1mm (pressure vent) and about 4mm (vented earmold). About 70% of patients with downward sloping hearing loss preferred the vented earmold, probably due to increased comfort. No association, however, was observed between the hearing loss configuration and the vent size of the earmold patients preferred.

Some authors have reported increased perception of tinnitus upon occlusion of the EAM, and have recommended vented earmolds for hearing aid adaptation.^14^, ^45^ We found no significant difference in NS scores for assessing annoyance due to tinnitus after using both vent sizes (p=0.96), which contradicted our initial hypothesis. The qualitative assessment of tinnitus showed no difference between both earmolds. Only 24% of cases showed different earmold performance; 12% improved with vented earmolds and 12% improved with pressure vents. Our findings are similar to those of Moura et al. (2004), who demonstrated that hearing aid features such as the model, technology and the presence and size of vents had no influence on tinnitus.^34^

Valente et al. (1996) also found that adaptation to hearing aids with vented earmolds favors hearing aid acceptance and reduce the discomfort associated with EAM occlusion, regardless of tinnitus.^46^ Kuk (1991) states that although the principal aim of amplification is to improve speech intelligibility, the patient’s subjective impression is fundamental for the acceptance and effective use of hearing aids.^44^ Nielsen (1975), however, found no significant difference in study groups regularly using hearing aids with vented and unvented earmolds, although the vented earmold group tended to use hearing aids with greater frequency.^43^

Although the benefits of vented earmolds are unquestionable, there are caveats such as an excessive escape of lower and middle frequencies, which may reduce the gain of hearing aids at these frequencies in patients with flat configuration hearing loss where low frequencies are also affected.^47^, ^48^ Analysis of the functional gain in the groups with flat configuration hearing loss showed significantly higher gains with the pressure vent at 500 and 1000 Hz (p=0.03). Small sized venting makes it possible to equalize air pressure without reducing the amplification of frequencies below 1000 Hz; air pressure balancing is only obtained by using vented earmolds.^10^

Possibly due to an excessive escape of lower frequencies, this same group tended to be less annoyed by tinnitus, as shown by the NS score for the pressure vent. Even so, 53.9% of these patients chose vented earmolds at the end of the trial. These findings are similar to those of Nielsen (1975), who also noted that 42.9% of patients in groups with the lowest thresholds at lower frequencies chose vented earmolds, explaining this choice as being due to greater comfort and improved sound quality.^43^

### Final comments

Although our findings did not confirm our original hypothesis that the vented earmold would be superior for reducing tinnitus in patients with hearing aids, our results indicate that significant relief may be obtained in patients with tinnitus (82%) and hearing loss (96%), which can positively affect the patient’s quality of life.

Furthermore, we were able to show that regardless of the auditory configuration of hearing loss, earmold venting may be fundamental for successful adaptation; 66% of patients preferred the vented earmold rather than the pressure vent, possibly due to increased comfort. On the other hand we confirmed the idea that larger vents may reduce the functional gain of hearing aids at certain frequencies. As such, individual audiometric characteristics should be taken into account when choosing an earmold type. Over half of the patients with flat configuration hearing loss preferred the vented earmold at the end of the trial, showing that from the patient’s point of view, comfort may be more important than auditory gain.

## CONCLUSION

82% o patients reported reduced annoyance due to tinnitus and 96% reported improvement of hearing loss by using hearing aids with at least one type of earmold. There was no statistically significant performance difference in the quantitative and qualitative assessments of both types of earmold.

Patients with a flat configuration hearing loss tended to present less annoyance from the tinnitus when using the pressure vent. Gain in function at 500 and 1000Hz was significantly higher with this type of earmold.

A positive correlation was seen between measurements of variation of annoyance due to tinnitus and hearing loss when using vented earmolds and pressure vents.
